# Dental implant bioactive surface modifications and their effects on osseointegration: a review

**DOI:** 10.1186/s40364-016-0078-z

**Published:** 2016-12-14

**Authors:** Hsiu-Wan Meng, Esther Yun Chien, Hua-Hong Chien

**Affiliations:** 1 Department of Periodontics, University of Texas School of Dentistry at Houston, Houston, TX USA; 2 College of Dentistry, The Ohio State University, Columbus, OH USA; 3 Division of Periodontology, College of Dentistry, The Ohio State University, 305 West 12th Avenue, Columbus, OH 43210 USA

**Keywords:** Dental implant, Bioactive surface modification, Osseointegration

## Abstract

**Background:**

The purpose of this article is to review and update the current developments of biologically active dental implant surfaces and their effect on osseointegration.

**Methods:**

PubMed was searched for entries from January 2006 to January 2016. Only in-vivo studies that evaluated the effects of biomolecular coatings on titanium dental implants inserted into the bone of animals or humans were included.

**Results:**

Thirty four non-review studies provided data and observations were included in this review. Within the criteria, four categories of biomolecular coatings were evaluated. The potential biomolecules include bone morphogenetic proteins in 8 articles, other growth factors in 8 articles, peptides in 5 articles, and extracellular matrix in 13 articles. Most articles had a healing period of 1 to 3 months and the longest time of study was 6 months. In addition, all studies comprised of implants inserted in animals except for one, which evaluated implants placed in both animals and humans. The results indicate that dental implant surface modification with biological molecules seem to improve performance as demonstrated by histomorphometric analysis (such as percentage of bone-to-implant contact and peri-implant bone density) and biomechanical testing (such as removal torque, push-out/pull-out tests, and resonance frequency analysis).

**Conclusions:**

Bioactive surface modifications on implant surfaces do not always offer a beneficial effect on osseointegration. Nevertheless, surface modifications of titanium dental implants with biomolecular coatings seem to promote peri-implant bone formation, resulting in enhanced osseointegration during the early stages of healing. However, long-term clinical studies are needed to validate this result. In addition, clinicians must keep in mind that results from animal experiments need not necessarily reflect the human clinical reality.

## Background

Osseointegrated implants have been used to replace missing teeth and as an anchorage for orthodontic tooth movement with direct bone contact [1]. The concept of osseointegration was originally introduced by Brånemark et al. [2] in 1969. Albrektsson et al. [3] suggested that this was “a direct functional and structural connection between living bone and the surface of a load carrying implant.” Another clinical definition provided by Zarb and Albrektsson [4] proposed that osseointegration was “a process whereby clinically asymptomatic rigid fixation of alloplastic materials is achieved and maintained in bone during functional loading.”

Surface modifications of titanium dental implants have been studied and applied to improve biological surface properties, which favors the mechanism of osseointegration. Machined implant surfaces, representing the starting point of implant surface design, were used for decades according to the classic protocols in which several months were essential to achieve osseointegration [5]. Consequently, topographic and chemical surface modifications replaced machined surfaces to a great extent in clinical application [6]. Various methods have been developed to increase the surface roughness of dental implants, including blasting with ceramic particles/acid etching [7, 8], titanium plasma spraying [9, 10], electrochemical anodization [11, 12], and calcium phosphate coatings [13, 14].

Furthermore, a novel research field investigating the addition of bioactive molecules to titanium implant surfaces has emerged in implant dentistry. A bioactive molecule is a material having an effect on or eliciting a response from living tissue [15]. Molecules with potential to be applied for bioactive purposes include bioceramics, ions, and biomolecules [16–19]. In this review article, the research interest was limited to titanium dental implants coated with a biomolecule, an organic molecule that is produced by a living organism.

Several reviews have been conducted to assess the effects of different implant surface modifications on peri-implant bone formation and osseointegration [20, 21]. Although it has been reported that the use of bioactive molecules represents a growing area of research in implant dentistry with an aim of achieving quicker osseointegration [22, 23], there are questions that have not previously been answered: (1) which type of biomolecular coatings are presently studied? and (2) what can we expect as the possible effects of biomolecular coatings? Consequently, these display the need for a broad review focused on current developments of biologically active dental implant surfaces. Therefore, the aim of this article is to review and update the current developments of biologically active dental implant surfaces and their impact on osseointegration.

## Materials and methods

### Criteria for Considering Studies for This Review

#### Inclusion criteria

- Current developments published during January 2006 to January 2016
- In vivo study
- Titanium implant surfaces coated with biomolecules
- Threaded screw-type implants or implants with recessions or indentations
- Evaluation of the effect of biomolecular coatings on bone formation or osseointegration

#### Exclusion criteria

- In vitro study
- Use of biomolecules to fill the defect before or after implant placement
- Cylindrical, non-threaded implants
- Intentional creation of peri-implant defects
- Evaluation of the effect of biomolecular coatings on soft tissue, regeneration, ridge augmentation or sinus floor elevation
- Implants that were loaded

### Search Methods for Identification of Studies

#### Electronic searching

PubMed (January 2006 to January 2016) was searched using the keywords “biologically active dental implant surface,” “bioactive dental implant surface,” “dental implant surface modification,” “bone morphogenetic proteins (BMPs),” “growth factor,” “peptide,” “Arg-Gly-Asp (RGD),” or “collagen.” Papers were limited to those articles published in English.

#### Hand searching

Reference lists of any potential studies were examined in an attempt to identify any other studies.

## Results

Among the available literature, 4 categories of biomolecular coatings were outlined and assessed in this review: (1) BMPs, (2) non-BMP growth factors, (3) peptides, and (4) extracellular matrix (ECM).

### Modification of dental implant surfaces with BMPs (Table 1)

Becker et al. [24] investigated bone formation onto sand-blasted and acid-etched (control group), chromosulfuric acid surface-enhanced (CSA group), and recombinant human BMP-2 (rhBMP-2) biocoated CSA [BMP-A group: non-covalently immobilized rhBMP-2 (596 ng/cm^2^); BMP-B group: covalently immobilized rhBMP-2 (819 ng/cm^2^)] implants after placement in the mandibles and tibiae of dogs. After 4 weeks of healing, bone to implant contact (BIC) values appeared to be highest for the BMP-B group, followed by BMP-A, CSA and the control in both the mandible and the tibia. Bone density measured at a distance of less than 1 mm adjacent to each implant revealed a similar pattern of difference between groups as to that of BIC; however, no differences between groups were observed at a distance greater than 1 mm. It was concluded that rhBMP-2 immobilized by covalent and noncovalent methods on CSA-treated implant surfaces appeared to be stable and stimulated direct bone apposition in a concentration-dependent manner.

**Table 1:** Surface modification of dental implants with BMPs

Author	Animal – bone site	Mode of surface modification	Length of study	Findings
Becker et al. [24]	Dog – mandible and tibia	1. C: sand-blasted and acid-etched 2. CSA 3. BMP-A: non-covalently immobilized rhBMP-2 (596 ng/cm^2^) 4. BMP-B: covalently immobilized rhBMP-2 (819 ng/cm^2^)	4 weeks	BIC and BD: BMP-B > BMP-A > CSA > C
Lan et al. [25]	Rabbit - femur	1. A: with rhBMP-2 2. B: without rhBMP-2	12 weeks	- Pull-out strength: A (36.5 N) > B (27.6 N) - Bone formation: A > B
Liu et al. [26]	Pig – maxilla	1. Ti + CaP + BMP-2 inc 2. Ti + CaP + BMP-2 ads 3. Ti + CaP + BMP-2 ads + inc 4. Ti + CaP 5. Ti + BMP-2 ads 6. Ti	3 weeks	- Bone volume was highest for Ti & CaP and lowest for CaP/BMP-2 ads - Bone-interface coverage of the implant surface was highest for CaP and lowest for Ti/BMP-2 ads
Wikesjo et al. [27]	Dog - mandible	1. TPO + rhBMP-2 (0.2 mg/ml) ads 2. TPO + rhBMP-2 (4.0 mg/ml) ads 3. TPO (control)	8 weeks	% of BIC: - TPO surfaces coated with 0.2 mg/ml rhBMP-2: 43.3% vs. TPO control: 71.7% - TPO surfaces coated with 4 mg/ml rhBMP-2: 35.4% vs. TPO control: 68.2%
Wikesjo et al. [28]	Monkey - maxilla	1. TPO + rhBMP-2 (2.0 mg/ml) ads 2. TPO + rhBMP-2 (0.2 mg/ml) ads 3. TPO (control)	16 weeks	% of BIC - Uncoated TPO surfaces: 74 - 75% - TPO surfaces coated with 2.0 mg/ml rhBMP-2: 43% - TPO surfaces coated with 0.2 mg/ml rhBMP-2: 37%
Huh et al. [29]	Dog - mandible	1. rhBMP-2 coated implants 2. uncoated anodized implants (control)	8 weeks	% of BIC - Uncoated control: 40.16% - rhBMP-2 coated: 41.88% Implant stability test - Uncoated control: ISQ = 74.27 - rhBMP-2 coated: ISQ = 79.21
Hunziker et al. [30]	Pig - maxilla	1. Ti 2. Ti + CaP 3. Ti + rhBMP-2 (10 μg) ads 4. Ti + CaP + rh BMP-2 (10 μg) ads 5. Ti + CaP + rh BMP-2 (12.95 μg) inc 6. Ti + CaP + rhBMP-2 ads + inc	1, 2, and 3 weeks	Volume fraction of total bone - Ti + CaP + BMP-2, with either ads or inc or both, have the highest values at 1 week - Ti + CaP + BMP-2 inc and Cap groups have the highest values at 2 weeks - Ti + CaP + BMP-2 inc and Cap groups have the highest values at 3 weeks
Kim et al. [31]	Dog - mandible	1. SLA (control) 2. SLA + rhBMP-2 (0.1 mg/mL) 3. SLA + rhBMP-2 (0.5 mg/mL) 4. SLA + rhBMP-2 (1 mg/mL)	8 weeks	% of BIC - Control: Buccal: 0.67%; Lingual: 23.37% - rhBMP-2 (0.1 mg/mL): Buccal: 10.24%; Lingual: 26.50% - rhBMP-2 (0.5 mg/mL): Buccal: 24.47%; Lingual: 35.45% - rhBMP-2 (1 mg/mL): Buccal: 18.42%; Lingual: 33.43% BV - Control: Buccal: 2.77%; Lingual: 46.50% - rhBMP-2 (0.1 mg/mL): Buccal: 13.30%; Lingual: 60.50% - rhBMP-2 (0.5 mg/mL): Buccal: 33.67%; Lingual: 66.17% - rhBMP-2 (1 mg/mL): Buccal: 35.67%; Lingual: 65.00% Implant stability test - Control: ISQ = 60.17 - rhBMP-2 (0.1 mg/mL): ISQ = 64.83 - rhBMP-2 (0.5 mg/mL): ISQ = 71.67 - rhBMP-2 (1 mg/mL): ISQ = 72.00

*CSA* chromosulfuric acid surface-enhanced; *BIC* bone to implant contact; *BD* bone density; *Ti* titanium; *CaP* calcium phosphate; *BMP* bone morphogenetic protein; *inc* incorporated; *ads* adsorbed; *TPO* titanium porous oxide; *SLA* sandblasted and acid-etched surface

Lan et al. [25] investigated the influence of rhBMP-2 on bone-implant osseointegration in the femurs of rabbits. Thirty-two implants were evenly divided into 2 groups. Implants in group A were coated with 1.0 mg rhBMP-2 and implants in group B were not coated with rhBMP-2. Twelve weeks after implantation, the pull-out binding strengths of group A were significantly greater than that of group B. Scanning electronic microscopy showed extensive mineralized matrixes on the surface of the implants in group A but not B. Under confocal laser scanning microscopy, there was significantly more percentage of marked bone adjacent to the implant surface in group A compared to group B at both 4 and 8 weeks. The authors concluded that rhBMP-2 improves the quantity and quality of implant-bone osseointegration.

In the maxillae of adult miniature pigs, Liu et al. [26] assessed the influence of BMP-2 and its mode of delivery on the osteoconductivity of dental implants with 6 different types of surfaces, including uncoated titanium (Ti) surfaces, BMP-2 adsorbed to uncoated Ti (Ti/BMP-2 ads), calcium phosphate (CaP)-coated Ti surfaces (CaPTi), BMP-2 adsorbed to CaPTi (CaPTi/BMP-2 ads), BMP-2 incorporated into CaPTi (CaPTi/BMP-2 inc), and BMP-2 adsorbed to and incorporated into CaPTi (CaP/BMP-2 ads + inc). After 3 weeks, the volume of bone deposited within the osteoconductive space was highest for coated and uncoated implants with no BMP-2, while the lowest value was achieved with coated implants bearing only adsorbed BMP-2. Bone coverage of the implant surface was highest for CaPTi, while the lowest amount of bone coverage was observed for Ti/BMP-2 ads. It was concluded that the osteoconductivity of implant surfaces can be negatively modulated by BMP-2 and its method of delivery.

Wikesjo et al. [27] studied whether adsorbing rhBMP-2 onto a titanium porous oxide (TPO) implant surface might increase or accelerate local bone formation and support osseointegration in the posterior mandible (type II bone) in dogs. Implants with a TPO surface were adsorbed with rhBMP-2 at 0.2 mg/ml or 4.0 mg/ml, whereas TPO implants without rhBMP-2 served as controls. After 8 weeks of healing, implants coated with rhBMP-2 (4.0 mg/ml) exhibited markedly increased bone formation and bone metabolic activity compared with that observed for implants coated with rhBMP-2 (0.2 mg/ml) and controls. BIC appeared significantly lower for rhBMP-2-coated implants when compared to the control, however, the BIC in BMP-2 coated implants is clinically respectable. Finally, it was concluded that rhBMP-2 adsorbed onto TPO implant surfaces is able to induce dose-dependent peri-implant bone remodeling, resulting in the formation of normal, physiologic bone and clinically applicable osseointegration within 8 weeks.

A similar study conducted by Wikesjo et al. [28] to evaluate local bone formation and osseointegration in the posterior maxillae (type IV bone) was analyzed in 8 adult monkeys. Each animal received three TPO implants adsorbed with either rhBMP-2 at 2.0 mg/ml or 0.2 mg/ml in one quadrant and three TPO implants without rhBMP-2 in the contra-lateral quadrant as control. After 16 weeks, BIC was significantly higher for uncoated TPO surfaces versus TPO surfaces coated with either 2 mg/ml or 0.2 mg/ml rhBMP-2. Additionally, rhBMP-2 coated TPO implants exhibited a pinpoint BIC pattern regardless of rhBMP-2 concentrations; on the other hand, the controls exhibited a thin layer of bone covering most of the implant threads, resulting in higher BIC. Sites receiving 2.0 mg/ml of rhBMP-2 implants exhibited new peri-implant bone formation, while contralateral controls exhibited residual native bone close to the implant surface. The authors concluded that rhBMP-2-coated TPO surfaces enhanced local bone formation in type IV bone in a dose-dependent fashion in non-human primates, resulting in significant osseointegration.

Huh et al. [29] investigated anodized implants coated with rhBMP-2 on the effect of bone formation in dogs. Eighteen uncoated and 18 rhBMP-2 coated implants were randomly installed into the mandibular alveolar ridge of 6 young adult dogs for 8 weeks. The implant stability quotient (ISQ) and histometric analysis were examined to evaluate the effect of rhBMP-2 on the stimulation of bone formation. The BMP group showed significantly higher ISQ values than the control group at 8 weeks after implant placement. Similarly, histometric analysis indicated that the changes of bucco-lingual alveolar bone level were significantly higher in the BMP group than in the control group. However, the differences in mean percentage of BIC and bone density were not statistically significant. It was concluded that the rhBMP-2 coated implants can enhance bone formation and increase implant stability in dogs.

In the maxillae of adult miniature pigs, Hunziker et al. [30] studied whether the capacity of BMP-2 to induce peri-implant bone formation can be influenced by its mode of delivery. Six different implant surfaces: (1) Ti implant surface as control; (2) CaP coated Ti implant; (3) BMP-2 (10 μg) adsorbed onto Ti implant; (4) BMP-2 (10 μg) adsorbed onto CaP coated Ti implant; (5) BMP-2 (12.95 μg) incorporated into CaP coated Ti implant; and (6) BMP-2 (10 μg + 12.95 μg) adsorbed onto and incorporated into CaP coated Ti implant were established and tested in the maxillae of 18 adult miniature pigs. The histomorphometric analysis of bone formation was at 1, 2, and 3 weeks after implant placement. The incorporated or adsorbed BMP-2 on CaP coated Ti implant surface did not benefit peri-implant bone formation compared to the CaP coated Ti implant surface. However, the osteoinductive efficacy of BMP-2 can be influenced by its mode of delivery.

A study was conducted to investigate the effects of rhBMP-2 on osseointegration in dogs [31]. Three different concentrations of rhBMP-2 (0.1, 0.5, and 1 mg/mL) were applied to sandblasted and acid etched (SLA) implants and served as an experimental group, while SLA implants were used as a control group. Two months after tooth extraction, four animals received implants coated with 3 different concentrations of rhBMP-2 in one side of the mandible while the contralateral site received SLA implants. BIC, bone volume (BV) and implant stability were analyzed at 8 weeks after implant placement. The mean BIC and BV were greater in the 0.5 and 1.0 mg/mL rhBMP-2 groups than in the 0.1 mg/mL and control groups. Furthermore, the ISQ values were highest in the 1.0 mg/mL group. It was concluded that the SLA implants coating with 0.5 and 1.0 mg/mL of rhBMP-2 was more effective in enhancing osseointegration.

### Modification of dental implant surfaces with non-BMP growth factors (Table 2)

Anitua [32] compared BIC of titanium implant surface (Ti) to human autologous plasma-rich growth factor-coated titanium implants (PRGF) in the tibiae and radii of goats. Histomorphometry analysis performed after 8 weeks showed that titanium implant surfaces with PRGF demonstrated significantly higher percentage of BIC. Every biopsy of the implants made with PRGF revealed bone surrounding the entire implant; whereas non-PRGF-coated implants were surrounded by cortical bone only in the middle third. Furthermore, 1391 implants were bioactivated with PRGF and installed in 295 patients. The author reported that 99.6% of the implants treated with PRGF were well osseointegrated. It was concluded that osseointegration was enhanced by covering the implant surface with PRGF before insertion into the alveolar bone.

Lan et al. [33] studied the effect of combining rhBMP-2 and recombinant human basic fibroblast growth factor (rhbFGF) or recombinant human insulin-like growth factor-1 (rhIGF-1) on bone-implant osseointegration in rabbits. Sixty-four implants were coated with polylactic acid and equally divided into 4 groups. Implants in group 1 were applied with 1.0 mg rhBMP-2 and 200 μg rhbFGF, group 2 with 1.0 mg rhBMP-2 and 250 μg rhIGF-1, group 3 with only 1.0 mg rhBMP-2, and group 4 were used as controls and had no growth factors applied. During the healing period, fluorescent bone markers were administered at 4 and 8 weeks. Twelve weeks after the implantation in the femurs of rabbits, implants and surrounding bone were collected and prepared for confocal laser scanning microscopy analysis. There was a statistical difference in bone formation between rhBMP-2 and non-rhBMP-2 groups at 4 or 8 weeks. The new bone formation in groups 1 and 2 was greater than that of group 3 at 8 weeks. It was concluded that rhBMP-2 could increase new bone formation and was able to act synergistically with rhbFGF and rhIGF-1 to improve osseointegration.

**Table 2:** Surface modification of dental implants with other growth factors

Author	Animal – bone site	Mode of surface modification	Length of study	Findings
Anitua [32]	Goat – tibia and radius	1. Ti (control) 2. Ti + PRGF	8 weeks	% of BIC - Ti + PRGF: 51% - Ti: 22%
Lan et al. [33]	Rabbit - femur	1. Group 1: rhBMP-2 (1.0 mg) + rhbFGF (200 μg) 2. Group 2: rhBMP-2 (1.0 mg) + rhIGF-1 (250 μg) 3. Group 3: rhBMP-2 (1.0 mg) 4. Group 4: no growth factor (control)	4 and 8 weeks	% of new bone formation - Group 1: 7.0% at 4 weeks, 10.0% at 8 weeks - Group 2: 7.6% at 4 weeks, 9.9% at 8 weeks - Group 3: 6.2% at 4 weeks, 8.0% at 8 weeks - Group 4: 5.0% at 4 weeks, 6.0% at 8 weeks
Park et al. [34]	Rabbit - tibia	1. Group 1: anodized under constant 300 voltage 2. Group 2: anodized under constant 300 voltage + FGF-FN fusion protein (65 μg/mL)	12 weeks	Mean removal torque value - Group 1: 37.6 Ncm - Group 2: 44.8 Ncm % of BIC - Group 1: 76.4% - Group 2: 88.0%
Nikolidakis et al. [35]	Goat – femoral condyle	1. Control: Ti 2. Ti-TGF(0.5): Ti + 0.5 μg TGF-β1 3. Ti-TGF(1.0): Ti + 1.0 μg TGF-β1	6 weeks	% of BIC - Ti: 65% - Ti-TGF(0.5): 48% - Ti-TGF(1.0): 45%
Schouten et al. [36]	Goat – Femoral condyle	1. Screw type implant: St 2. St + CaP 3. St + CaP + TGF-β1 (1.0 μg) 4. Push-in type implant: Pi 5. Pi + CaP 6. Pi + CaP + TGF-β1 (1.0 μg)	12 weeks	% of BIC - St: 44% - St + CaP: 58% - St + CaP + TGF-β1: 64% - Pi : 23% - Pi + CaP: 46% - Pi + CaP + TGF-β1: 52%
Lee et al. [37]	Rabbit – tibia	1. Group 1: anodized (under 300 voltage) 2. Group 2: anodized + 0.02 ml PLGA 3. Group 3: anodized + 0.02 ml PLGA/bFGF (10 ng bFGF) 4. Group 4: anodized + 0.2 ml PLGA/bFGF (100 ng bFGF)	12 weeks	% of BIC - Groups 1: 31.4% - Group 2: 33.6% - Group 3: around 37% - Group 4: 44.7%
Ramazanoglu et al. [38]	Pig – frontal skull	1. AE (control) 2. CaP coated 3. CaP + rhBMP-2 inc 4. CaP + rhVEGF_I65_ inc 5. CaP + rhBMP-2 inc + rhVEGF_I65_ inc	1, 2, and 4 weeks	% of BIC for ROI 1 + 4 - AE: 3.4% at 1 week; 11.5% at 2 weeks; 16.1% at 4 weeks - CaP: 5.2% at 1 week; 19.2% at 2 weeks; 23.5% at 4 weeks - CaP + BMP-2: 1.8% at 1 week; 15.5% at 2 weeks; 21.6% at 4 weeks - CaP + VEGF: 3.1% at 1 week; 15.2% at 2 weeks; 22.6% at 4 weeks - CaP + BMP-2 + VEGF: 3.3% at 1 week; 22.1% at 2 weeks; 23.2% at 4 weeks % of BIC for ROI 2 + 3 - AE: 10.6% at 1 week; 14.6% at 2 weeks; 20.1% at 4 weeks - CaP: 15.4% at 1 week; 22.6% at 2 weeks; 24.9% at 4 weeks - CaP + BMP-2: 8.2% at 1 week; 27.3% at 2 weeks; 23.6% at 4 weeks - CaP + VEGF: 8.5% at 1 week; 23% at 2 weeks; 24.4% at 4 weeks - CaP + BMP-2 + VEGF: 9.6% at 1 week; 31.7% at 2 weeks; 31.8% at 4 weeks
Schliephake et al. [39]	Rat - tibia	1. SAE (control) 2. SAE + OAS 3. SAE + OAS + rhVEGF-conjugated to complementary oligonucleotide strands	1, 4, and 13 weeks	% of BIC for 1 and 4 weeks - SAE: 14.5% at 1 week; 40.6% at 4 weeks - SAE + OAS: 14.1% at 1 week; 40.2% at 4 weeks - SAE + OAS + VEGF: 15.1% at 1 week; 60.1% at 4 weeks % of BIC for 13 weeks All groups were between 75% to 80% with no significantly differences between groups

*Ti* titanium implant surface; *BIC* bone to implant contact; *PRGF* human plasma-rich growth factor; *hrBMP-2* recombinant human bone morphogenetic protein-2; *rhbFGF* recombinant human basic fibroblast growth factor; *rhIGF-1* recombinant human insulin-like growth factor; *FGF-FN* fibroblast growth factor-fibronectin; *TGF-β1* transforming growth factor β1; *St* screw type implant; *Pi* push-in type implant; *CaP* calcium phosphate coating; *PLGA* poly(lactide-co-glycolide); *bFGF* basic fibroblast growth factor; *AE* acid-etched; *VEGF* vascular endothelial growth factor; *CaP* calcium phosphate; *SAE* sandblasted and acid-etched; *OAS* DNA oligonucleotide anchor strands

In a rabbit tibia model, Park et al. [34] evaluated the bone response around anodized titanium implants coated with fibroblast growth factor-fibronectin (FGF-FN) fusion protein. Twenty implants were evenly divided into 2 groups. Implants in group 1 were anodized under a constant voltage of 300 V, whereas implants in group 2 were anodized under a constant voltage of 300 V and then soaked in a solution containing FGF-FN (65 μg/mL) for 24 h. After 12 weeks of healing, the mean removal torque value for group 2 was significantly greater than that of group 1. Under histomorphometric analysis, the percentage of BIC was significantly higher in group 2 than in group 1. The authors concluded that the FGF-FN fusion protein coating on anodized implants may enhance osseointegration.

Nikolidakis et al. [35] examined the effect of transforming growth factor β1 (TGF-β1) on the early bone healing around dental implants installed into the femoral condyle of goats. Eight healthy female goats were used in this study, and each animal received 3 implants: one Ti (control), one Ti loaded with 0.5 μg TGF-β1 (Ti-TGF_0.5_), and one Ti loaded with 1.0 μg TGF-β1 (Ti-TGF_1.0_). Six weeks after implantation, an intervening fibrous tissue layer occurred around approximately half of the TGF-β1 loaded implants. Ti implants without TGF-β1 treatment demonstrated the highest percentage of BIC, while implants with 1.0 μg TGF-β1 showed the lowest amount. The difference between the two aforementioned groups was statistically significant. The authors concluded that a low dose of TGF-β1 has a negative influence on the integration of oral implants in trabecular bone during the early post-implantation healing phase.

Schouten et al. [36] investigated the effect of implant design, surface properties, and TGF-β1 on peri-implant bone response. Two geometrically different implant types, screw type (St) and push-in type (Pi), were used. Additionally, the implant surfaces were modified with an electrosprayed CaP coating, either enriched or not enriched with TGF-β1. A total of 54 implants, equally divided into six groups, were installed into the femoral condyles of nine goats for 12 weeks. With respect to implant geometry, St implants showed an overall better biological healing process than Pi implants. In term of surface properties, the deposition of CaP coating significantly increased percentage of BIC for both implant types. The enrichment of CaP-coated implants with TGF-β1, however, did not significantly improve peri-implant bone response. The authors concluded that an extensive improvement of the bone response to titanium implants can be obtained by adding an electrosprayed CaP coating. The supplementation of a 1 μg TGF-β1 coating has only a marginal effect.

Lee et al. [37] analyzed the effect of poly(lactide-co-glycolide) (PLGA) in combination with bFGF coating on an anodized titanium implant surface by electrospray. Forty-eight implants were equally divided into 4 groups and inserted into rabbit tibiae. Group 1 implants were anodized under 300 V; group 2 implants were anodized and then coated with 0.02 ml PLGA; group 3 implants were anodized and then coated with 0.02 ml PLGA/bFGF (10 ng bFGF); and group 4 implants were anodized and then coated with 0.2 ml PLGA/bFGF (100 ng bFGF). Histomorphometric analysis was performed via light microscopy and computerized image analysis of the implants that had been in situ for 12 weeks. Implants in group 4 (44.7%) had significant higher mean BIC percentage than that seen in groups 1 (31.4%) and 2 (33.6%). It was suggested that coating a titanium implant with PLGA incorporated with bFGF by electrospray may enhance bone formation near the surface of an implant installed in bone.

Ramazanoglu et al. [38] studied whether coating implant surfaces with rhBMP-2 and recombinant human vascular endothelial growth factor I65 (rhVEGFI65) affects osseointegration in pigs. Five different groups of implants: (1) acid-etched surface (AE; control group); (2) CaP coated surface (CaP group); (3) CaP bearing incorporated rhBMP-2 (BMP group); (4) CaP bearing incorporated rhVEGFI65 (VEGF group); and (5) CaP bearing incorporated rhBMP-2 + rhVEGFI65 (BMP + VEGF group) were established and tested in the frontal skulls of 9 pigs. Osseointegration was assessed by histomorphometric analysis of bone formation at 1, 2, and 4 weeks. At 2 weeks, the BMP and BMP + VEGF groups showed significant enhancement in BV density compared to the AE control group. All implants with CaP coating demonstrated significant enhanced BIC rates compared with the AE controls at 2 weeks. However, the BMP + VEGF group did not significantly enhance BIC at 4 weeks. It was concluded that the biomimetic CaP coated implant surfaces with both BMP and VEGF enhances BV density, but not BIC.

Schliephake et al. [39] tested whether rhVEGF stimulates peri-implant bone formation in rats. Three different implant surfaces: (1) sandblasted acid-etch (SAE) implants; (2) SAE implants coated with DNA oligonucleotide anchor strands (SAE + OAS); and (3) SAE + OAS hybridized with rhVEGF-conjugated to complementary oligonucleotide strands, were studied in the tibiae of 36 rats. The BIC and BD were assessed 1, 4, and 13 weeks after implant placement. Implant surfaces with rhVEGF hybridization showed the highest BIC 1 month after implant placement. It was concluded that rhVEGF can accelerate BIC to a certain extent.

### Modification of dental implant surfaces with peptides (Table 3)

Germanier et al. [40] investigated whether implant surfaces with Arg-Gly-Asp (RGD)-peptide-modified polymer could enhance early bone apposition in the maxillae of miniature pigs. Test and control implants had the same microrough structure identical to SLA surfaces, but differed in their surface chemistry coating. Four different implant surfaces: (1) SLA without coating as control; and coating with (2) poly(L-lysine)-graft-poly(ethylene glycol) (PLL-*g*-PEG); (3) PLL-*g*-PEG/PEG-RGD; or (4) PLL-*g*-PEG/PEG-Arg-Asp-Gly(RDG) were examined histomorphometrically for osteoblastic activity and bone apposition. The RGD-modified implants demonstrated significant increase in BIC apposition at 2 weeks as compared with the controls. However, no further significant increase in percentage of BIC was observed in RGD-coated implants at 4 weeks as compared to the controls. On the contrary, the PEG and PEG/RDG groups did show significantly higher BIC than the control group at 4 weeks. It was concluded that the PLL-*g*-PEG/PEG-RGD coatings may stimulate greater bone apposition in the very early stages of bone healing following implant placement.

**Table 3:** Surface modification of dental implants with peptides

Author	Animal – bone site	Surface modification	Length of study	Findings
Germanier et al. [40]	Pig – maxilla	1. SLA (control) 2. SLA + PLL-*g*-PEG 3. SLA + PLL-*g*-PEG/PEG-RDG 4. SLA + PLL-*g*-PEG/PEG-RGD	2 and 4 weeks	% of BIC - SLA: 43.6% at 2 weeks, 62.5% at 4 weeks - SLA + PLL-*g*-PEG: 55.9% at 2 weeks, 67.4% at 4 weeks - SLA + PLL-*g*-PEG/PEG-RDG: 48.5% at 2 weeks, 75.5% at 4 weeks - SLA + PLL-*g*-PEG/PEG-RGD: 61.7% at 2 weeks, 62.5% at 4 weeks
Barros et al. [41]	Dog – mandible	1. A: microstructured + HA + low concentrated (20 μg/ml) peptide 2. B: microstructured + HA 3. C: microstructured 4. D: microstructured + HA + high concentrated (200 μg/ml) peptide	12 weeks	% of BIC - A: 60.4% - B: 62.4% - C: 58.0% - D: 65.4% BD - A: 54.7% - B: 46.0% - C: 45.3% - D: 40.7%
Yang et al. [42]	Rabbit – femur and tibia	1. Control: uncoated 2. RGD-coated	4, 8, and 12 weeks	% of BIC - Control: 50.2%, 58.5%, and 60.9% at 4, 8, and 12 weeks, respectively - RGD-coated: 70.0%, 74.9%, and 82.2% at 4, 8, and 12 weeks, respectively
Lutz et al. [43]	Pig – forehead	1. Group A: HA 2. Group B: HA + P-15 (20 μg/ml) 3. Group C: HA + P-15 (200 μg/ml)	14 and 30 days	% of BIC for ROI A and ROI B, respectively - A: 63.8 & 76.7% at 14 days, 70.9 & 75.8% at 30 days - B: 72.1 & 72.5% at 14 days, 69.9 & 74.6% at 30 days - C: 88.2 & 88.7% at 14 days, 80.5 & 88.5% at 30 days BD for ROI A and ROI B, respectively - A: 49.5 & 36.2% at 14 days, 38.4 & 28.7% at 30 days - B: 44.0 & 31.6% at 14 days, 45.7 & 42.9% at 30 days - C: 44.9 & 36.1% at 14 days, 47.2 & 44.5% at 30 days
Yoo et al. [44]	Rabbit - tibia	1. Anodized Ti implant (control) 2. PLGA/rhBMP-2 coated	3 and 7 weeks	% of BIC (total) - Control: 27.26% at 3 weeks; 31.47% at 7 weeks - PLGA/rhBMP-2: 34.69% at 3 weeks; 35.32% at 7 weeks

*SLA* sandblasted and acid-etched surface; *PLL-g-PEG* poly(L-lysine)-graft-poly(ethylene glycol); *RDG* Arg-Asp-Gly; *RGD* Arg-Gly-Asp; *BIC* bone to implant contact; *BD* bone density; *HA* hydroxyapatite; *P-15* P-15 peptide; *ROI* region of interest

Barros et al. [41] analyzed the effect of a biofunctionalized implant surface on osseointegration in the mandibles of dogs for 12 weeks. All implants were lined by the same microrough surface topography provided by the grit-blasting/acid-etching process to create a micro-structured surface. For biofunctionalization of the implant surface, a low or high concentration of bioactive peptide was absorbed to hydroxyapatite (HA) coatings. Thus, 4 different types of implant surfaces were tested: (1) micro-structured + HA + a low concentration of bioactive peptide (20 μg/mL); (2) micro-structured + HA; (3) micro-structured; and (4) micro-structured + HA + a high concentration of bioactive peptide (200 μg/mL). Implants with 200 μg/mL peptide had the highest mean value of direct BIC, but no statistically significant differences were detected between the groups. In addition, bone density analysis revealed that implant surfaces with 20 μg/mL peptide provided a higher adjacent bone density when compared to the other groups. Nevertheless, the differences between the groups were also not statistically significant. The authors concluded that biofunctionalization of the implant surface might interfere in the bone apposition around implants, especially regarding the aspect of bone density.

Yang et al. [42] compared the ability of uncoated titanium implant surfaces with RGD-coated surfaces to bond to bone in the femurs and tibiae of rabbits. Sixty implants (30 uncoated control and 30 RGD-coated) were inserted into the femurs of 30 rabbits, and 30 implants (15 control and 15 RGD-coated) were installed into the tibias of 15 rabbits. The RGD-coated implants exhibited significantly greater percentages of BIC than those of the control implants at 4, 8, and 12 weeks after implantation. In addition, the RGD-coated implants revealed statistically significant higher removal torque values at 8 and 12 weeks. It was concluded that RGD-coated implants have a positive effect on bone-bonding ability by producing higher BIC and removal torque values.

Lutz et al. [43] studied the influence of a P-15 peptide coated implant on early implant osseointegration in adult pigs for a period of 14 and 30 days. All surfaces of dental implants were grit-blasted and acid-etched followed by HA coating. For the implants in the test group, two concentrations of P-15 (20 μg/ml and 200 μg/ml) were bound to the HA surface. Implants without P-15 coating served as the control group. Implants with the 200 μg/ml P-15 had significantly higher value of BIC at 14 and 30 days compared with the other groups. Both concentrations of P-15 revealed better peri-implant bone density compared to the control group at 30 days. It was concluded that biofunctionalization of the implant surface with a biomimetic active peptide has a positive effect on osseointegration by creating higher percentages of BIC at 14 and 30 days and greater peri-implant bone density at 30 days.

Yoo et al. [44] examined whether biochemical coating of anodized Ti implant surface with poly(lactide-co-glycolide) (PLGA) and BMP-2 stimulates bone growth in rabbits. Eighteen of each group – anodized Ti implants (control group) and implants coated with 80 μL of PLGA and 50 μg/mL of rhBMP-2 (experimental group) – were placed in the tibiae of 8 rabbits for 3 and 7 weeks. Under histomorphometric analysis, the BIC was significantly higher in the PLGA/BMP-2 group than those of the control group at 3 weeks. However, the significant difference disappeared at 7 weeks. The authors concluded that PLGA/BMP-2 coated implants facilitated osseointegration during early healing.

### Modification of dental implant surfaces with ECM (Table 4)

Morra et al. [45] investigated whether biochemical modification of the anodized titanium surfaces with collagen (ColpTi) has a positive effect on osseointegration in rabbit femur trabecular bone. Histomorphometric analysis revealed that a higher amount of bone inside the screw threads and in contact with the surface was observed on ColpTi implants at 4 weeks. ColpTi had a significantly higher percentage of BIC compared to the control anodized titanium surface. They concluded that osseointegration in trabecular bone can be enhanced by biochemical surface modification with collagen.

**Table 4:** Modification of dental implant surfaces with ECM

Author	Animal – bone site	Surface modification	Length of study	Findings
Morra et al. [45]	Rabbit – femur	1. pTi: anodization of titanium implant 2. ColpTi: collagen coated titanium implant	4 weeks	% of BIC - pTi: 36.9% - ColpTi: 63.7% % of bone ingrowth - pTi: 29.0% - ColpTi: 43.3%
Schliephake et al. [46]	Dog - mandible	1. Machined titanium surface (control) 2. Machine + Collagen I (collagen only) 3. Machine + calcium phosphate (HA-only) 4. Machine + calcium phosphate + Collagen I (composite)	1 and 3 months	% of BIC - Control: 31.5% at 1 month, 41.2% at 3 months - Collagen-only: 41.9% at 1 month, 60.2% at 3 months - HA-only: 45.2% at 1 month, 61.7% at 3 months - Composite: 62.6% at 1 month, 59.0% at 3 months BVD - Control: 16.1% at 1 month, 40.6% at 3 months - Collagen-only: 22.9% at 1 month, 62.6% at 3 months - HA-only: 33.0% at 1 month, 58.5% at 3 months - Composite: 40.9% at 1 month, 67.3% at 3 months
Stadlinger et al. [47]	Pig – mandible	1. Coll: collagen type I 2. Coll/CS: collagen type I + chondroitin sulfate 3. Coll/CS/BMP: collagen type I + chondroitin sulfate + BMP-4	22 weeks	% of BIC - Coll: 45% - Coll/CS: 55% - Coll/CS/BMP: 41% Implant stability test at 22 weeks - Coll: ISQ = 73 - Coll/CS: ISQ = 69 - Coll/CS/BMP: ISQ = 67
Ferguson et al. [48]	Sheep – pelvis	1. Ti: sandblasted and acid-etched titanium 2. Zir: sandblasted and etched zirconia 3. CaP: Ti coated with calcium phosphate 4. APC: Ti modified via anodic plasma-chemical treatment 5. Bisphos: bisphosphonate coated Ti 6. Coll/CS: Ti coated with collagen containing chondroitin sulfate	8 weeks	Removal torque values at 8 weeks (N-mm) - Ti: 1,884 - Zir: 1,005 - CaP: 1,683 - APC: 919 - Bisphos: 1,835 - Coll/CS: 1,593
Stadlinger et al. [49]	Pig – mandible	1. Coll: collagen coated Ti 2. Coll/DC: collagen + DC 3. Coll/CS: collagen + CS 4. Coll/DC/TGF: collagen + DC + TGF- β1 5. Coll/CS/BMP: collagen + CS + BMP-4 6. Coll/DC/CS/BMP/TGF: collagen + DC + CS + BMP-4 + TGF- β1	3, 4, 5 and 6 weeks	% of BIC at 3, 4, 5, and 6 weeks Total - Coll: 12.45% - Coll/DC: 12.65% - Coll/CS: 28.28% - Coll/DC/TGF: 16.27% - Coll/CS/BMP: 22.92% - Coll/DC/CS/BMP/TGF: 11.43%
Stadlinger et al. [50]	Pig – mandible	1. Coll: collagen 2. Coll/CS: collagen + CS 3. Coll/CS/BMP-4: collagen + CS + rhBMP-4	6 months	% of BIC - Coll: 30% - Coll/CS: 40% - Coll/CS/rhBMP-4: 27%
Langhoff et al. [51]	Sheep – pelvis	1. Control: sandblasted and acid-etched Ti 2. CaP: calcium phosphate coated 3. APC: plasma anodized 4. Coll/CS: collagen + CS coated 5. Bisphos: bisphosphonate coated 6. Zir: sandblasted and etched zirconia	8 weeks	- All implants were well osseointegrated - No significant differences in % of BIC
Schliephake et al. [52]	Dog – mandible	1. MS: machined surface 2. DAE: dual acid-etched surface 3. RGD: RGD + DAE 4. Coll: collagen I + DAE 5. Coll/CS: collagen I + CS + DAE 6. Coll/CS/BMP-2: collagen I + CS + BMP-2 + DAE	4 and 12 weeks	% of BIC - MS: 25.4% at 4 weeks, 37.7% at 12 weeks - DAE: 40.9% at 4 weeks, 57.6% at 12 weeks - RGD: 41.4% at 4 weeks, 59.4% at 12 weeks - Coll: 40.4% at 4 weeks, 56.3% at 12 weeks - Coll/CS: 31.1% at 4 weeks, 66.4% at 12 weeks - Coll/CS/BMP-2: 43.7% at 4 weeks, 60.6% at 12 weeks
Stadlinger et al. [53]	Pig – mandible	1. Control: sandblasted and acid-etched Ti 2. Coll/CS1: Ti + collagen + low dose CS 3. Coll/CS2: Ti + collagen + high dose CS	1 and 2 months	% of BIC for the entire implant at 1 and 2 months - Control: 51.6% at 1 month, 62.7% at 2 months - Coll/CS1: 68.4% at 1 month, 71% at 2 months - Coll/CS2: 63.1% at 1 month, 67.9% at 2 months
Morra et al. [54]	Rabbit – femur and tibia	1. Ti: acid-etched titanium 2. CollTi: collagen type I + Ti	2 and 4 weeks	% of BIC was significantly higher around CollTi when compared with Ti both in femur and tibia at 2 weeks. No significant differences were observed at 4 weeks.
Alghamdi et al. [55]	Dog - mandible	1. Ti (control) 2. Nano-CaP-coated 3. Type I collagen coated	4 and 12 weeks	Overall BV - Ti: 47.5% at 4 weeks; 65.1% at 12 weeks - Nano-CaP: 49.5% at 4 weeks; 67.2% at 12 weeks - Collagen: 61.4% at 4 weeks; 72.7 at 12 weeks Inner zone BV - Ti: 10.5% at 4 weeks; 37.5% at 12 weeks - Nano-CaP: 23.1% at 4 weeks; 38.2% at 12 weeks - Collagen: 28.4% at 4 weeks; 52.4 at 12 week
Stadlinger et al. [56]	Pig - maxilla	1. SAE 2. Collagen type I coating (Coll) 3. Coll + CS (low amount) 4. Coll + CS (high amount) 5. Coll + high-SH 6. Coll + low-SH	4 and 8 weeks	% of BIC - SAE: 56% at 4 weeks; 66.4% at 8 weeks - Coll: 36.8% at 4 weeks; 68.7% at 8 weeks - Coll + CS (low): 70.4% at 4 weeks; 74.7% at 8 weeks - Coll + CS (high): 51.2% at 4 weeks; 63.5% at 8 weeks - Coll + high-SH: 50.3% at 4 weeks; 64.3% at 8 weeks - Coll + low-SH: 53.9% at 4 weeks; 65.5% at 8 weeks BVD - SAE: 23.2% at 4 weeks; 45.2% at 8 weeks - Coll: 26.2% at 4 weeks; 48.9% at 8 weeks - Coll + CS (low): 28.1% at 4 weeks; 54.9% at 8 weeks - Coll + CS (high): 27.4% at 4 weeks; 45.2% at 8 weeks - Coll + high-SH: 23.6% at 4 weeks; 42.9% at 8 weeks - Coll + low-SH: 21.8% at 4 weeks; 46.9% at 8 weeks
Lee et al. [57]	Rabbit - tibia	1. AE (control) 2. HA coating 3. Coll + HA coating 4. Coll + HA + BMP-2 coating	6 weeks	% of BIC - AE: 21.38% - HA coating: 24.18% - Coll + HA coating: 41.45% - Coll + HA + BMP-2 coating: 30.72%

*ECM* extracellular matrix; *HA* hydroxyapatite; *BVD* newly formed peri-implant bone; *BMP-4* bone morphogenetic protein-4; *Ti* titanium implant; *ISQ* implant stability quotient; *CaP* calcium phosphate; *Bisphos* bisphosphonate; *CS* chondroitin sulfate; *DC* decorin; *TGF-β1* transforming growth factor β1; *rhBMP-4* recombinant human bone morphogenetic protein-4; *RGD* RGD peptide; *SH* sulfate hyaluronan; *AE* acid-etched

Schliephake et al. [46] examined whether a composite coating of calcium phosphate and mineralized collagen I could enhance peri-implant bone formation. Four types of implants were evaluated in the mandibles of dogs for 3 months by measuring BIC and BV density (BVD). The control group comprised of implants with an uncoated machined titanium surface. The modified groups included implants coated with HA, collagen I, or a composite coating of HA and mineralized collagen I. After 1 month, the percentage of BIC was significantly higher only in the group of implants with the composite coating of calcium phosphate and mineralized collagen I. All coated implants showed significantly higher BVD of newly formed peri-implant bone after 1 month. Both BIC and BVD were significantly greater in all coated implants compared to the controls after 3 months. The authors concluded that coating an implant by biomimetically combining collagen I and HA can enhance BIC and peri-implant bone formation.

Stadlinger et al. [47] studied whether the application of chondroitin sulfate in combination with type I collagen, with or without BMP-4, can augment ossification and thus improve implant stability. One hundred and twenty implants were placed in the mandibles of 20 miniature pigs for 6 months. Three different surface coatings were produced: (1) collagen type I (coll); (2) coll/chondroitin sulfate (coll/CS); and (3) coll/CS/BMP-4 (coll/CS/BMP). Six implants (2 of each type of surface) were randomly installed on either side of the mandible in each minipig. The histomorphometry results demonstrated that the percentage of BIC was highest for coll/CS, followed by coll and coll/CS/BMP. The implant stability, as measured by resonance frequency analysis (RFA), was highest in the coll group. Although statistically significant differences were not observed in both BIC and RFA, coll and coll/CS implant coatings had a positive trend in beneficial characteristics for osseointegration, compared with the further integration of BMP-4.

Using a sheep pelvis model, Ferguson et al. [48] tested 6 different implant surface modifications and their effect on implant osseointegration at 2, 4, and 8 weeks after implantation. Peri-implant bone density and removal torque were analyzed on 6 different implant surfaces: (1) sandblasted and acid-etched titanium (Ti), (2) sandblasted and acid-etched zirconia, (3) Ti coated with calcium phosphate (CaP), (4) Ti modified via anodic plasma-chemical treatment (APC), (5) bisphosphonate-coated Ti (Bisphos), and (6) Ti coated with collagen containing chondroitin sulfate (Coll/CS). No significant differences in peri-implant bone density were identified between groups. Removal torque values for Ti, CaP, Bisphos and Coll/CS were significantly higher than those for zirconia and APC at 8 weeks after implant placement. The authors suggested that biofunctional coating of the implant surface with calcium phosphate, bisphosphonate or collagen containing chondroitin sulfate seems to have the potential to enhance peri-implant bone healing, but no additional benefit can be shown when compared to the effectiveness of SLA surface modification.

Stadlinger et al. [49] examined whether implants coated with extracellular matrix (ECM) components could improve osseointegration in pigs. The 6 surface coatings tested in this study were: (1) collagen (coll); (2) collagen and decorin (coll/DC); (3) collagen and chondroitin sulfate (coll/CS); (4) coll/DC and TGF-β1 (coll/DC/TGF); (5) coll/CS and BMP-4 (coll/CS/BMP); and (6) coll/CS/DC, TGF-β1 and BMP-4 (coll/CS/DC/BMP/TGF). Eight miniature pigs each received 6 implants in the mandible. The highest BIC was observed in coll/CS or coll/CS/BMP coated implants during the 6 weeks of healing, and was statistically higher at weeks 5 and 6 compared to other coatings. The authors implied that implant surfaces coated with coll/CS and coll/CS/BMP could lead to a higher degree of bone formation compared to other ECM components.

Stadlinger et al. [50] tested whether the addition of chondroitin sulfate (CS) and rhBMP-4 on a collagen-coated (coll) implant could further improve osseointegration in pigs. One hundred and twenty implants with 3 distinct surface coatings (coll, coll/CS, and coll/CS/rhBMP-4) were placed into the mandibles of 20 minipigs. The percentage of BIC was measured 6 months after implantation by histomorphometric analysis. The highest percentage of BIC was observed in coll/CS (40%), followed by coll (30%) and coll/CS/rhBMP-4 (27%). The authors suggested that the addition of CS to a collagen coated implant could promote osseointegration. However, further inclusion of a fairly low amount of rhBMP-4 had a negative effect on bone formation compared to coll/CS.

Langhoff et al. [51] compared 6 modified implant surfaces for osseointegration in a sheep pelvis model for 8 weeks. The six types of dental implant surfaces tested in this study were: (1) sandblasted and acid-etched (control), (2) calcium phosphate (CaP), (3) plasma anodized (APC), (4) type I collagen + chondroitin sulfate (coll/CS), (5) bisphosphonate (BisP), and (6) zirconia (Zr). All tested implants revealed decent osseointegration with only slight differences compared to the control implant surfaces. New bone formation was observed around all tested implants in the cancellous bone by 2 weeks and built up steadily until 8 weeks. The authors concluded that there were no significant differences in osseointegration between the six different implant surfaces. However, there was a clear tendency for col/CS, CaP and BisP to show better BIC values at 8 weeks compared to APC or Zr.

In the mandibles of foxhounds, Schliepake et al. [52] evaluated whether organic coatings on an implant surface could enhance peri-implant bone formation. Six types of implants were examined histomorphometrically in each foxhound: (1) machined surface (MS), (2) dual acid-etched surface (DAE), (3) DAE coated with RGD (RGD), (4) DAE coated with type I collagen (coll), (5) DAE coated with coll and chondroitin sulfate (coll/CS), and (6) coll/CS and rhBMP-2 (coll/CS/BMP-2). After 1 month, all coated implant groups exhibited significantly higher BIC values than that of the MS group, but not from that of the DAE group. After 3 months, with the exception of the coll group, the same held true for the mean BIC of all coated DAE groups. The mean BVD of the newly formed peri-implant bone did not reach a significant difference among any of the surfaces in 1 and 3 months. The authors concluded that organic coatings with coll, RGD, or CS with or without rhBMP-2 on DAE implant surface did not enhance peri-implant BV density when compared with the DAE surfaces, but did improve BIC when compared with the MS surfaces. It appears that the organic coatings did not offer further benefits than the benefits of DAE surfaces.

Stadlinger et al. [53] investigated whether coating an implant surface with collagen and chondroitin sulfate would enhance bone formation and implant stability, compared with an uncoated control. Three different implant surfaces, (1) sandblasted acid-etched implants (control), (2) collagen with low dose chondroitin sulfate (CS1), and (3) collagen with high dose chondroitin sulfate (CS2), were tested in the mandibles of 20 minipigs. Bone formation was determined by the percentage of BIC and relative peri-implant BV density (rBVD) after 1 and 2 months of healing, whereas implant stability was measured by RFA. Coated implants had significantly higher BIC compared with controls after 1 month of healing, but no significant difference could be found between CS1 and CS2. The percentage of BIC was increased for all surfaces after 2 months of healing; however, no significant differences were identified among the three groups. Similarly, no statistical differences in rBVD and RFA were found. It was concluded that collagen/CS has an encouraging effect on bone formation in short-term healing.

Morra et al. [54] investigated the effect of collagen covalently linked to acid-etched implants on peri-implant bone formation. Implants were installed in rabbit femurs and tibiae for up to 4 weeks. Histomorphometric analysis at 2 weeks revealed that significantly higher BIC was observed in collagen coated implants both in femurs and tibiae. On the other hand, no significant differences in BIC were detected at 4 weeks. The authors concluded that type I collagen covalently linked to acid-etched Ti implant surfaces could enhance peri-implant bone formation during early healing.

In the mandibles of dogs, Alghamdi et al. [55] evaluated whether type I collagen improves peri-implant bone formation. Three types of implants: (1) non-coated Ti implants; (2) nano-CaP-coated Ti implants; and (3) type I collagen-coated implants were placed in the mandibles of 16 beagle dogs 3 months after tooth extraction. Both histomorphometry and micro-CT were measured to evaluate peri-implant bone formation at 4 and 12 weeks after implant placement. Three different zones (inner: 0-300 μm; middle: 300-600 μm; and outer: 600-1000 μm) of bone around the implant surfaces were assessed. Nano-CaP and collagen-coated implants demonstrated a significantly higher BV in the inner zone compared to non-coated implants at 4 weeks. However, no significant difference was identified in the BV between all groups at 12 weeks as indicated by both histomorphometric and micro-CT analysis. It was concluded that collagen modification of implant surfaces did not improve peri-implant bone formation.

Stadlinger et al. [56] evaluated whether extracellular matrix coating on implant surfaces increases bone formation in minipigs. Six different implant surfaces were created and tested in the maxillae of 20 minipigs: (1) SAE Ti implant; (2) collagen type I coated implants; (3) collagen type I + low amount of CS ; (4) collagen type I + high amount of CS; (5) collagen type I + high-sulfate hyaluronan; and (6) collagen type I + low-sulfate hyaluronan. The BIC and BV density were measured using histomorphometric analysis at 4 and 8 weeks after implant placement. ISQ values were obtained for implant stability. Implants coated with the lower CS and collagen showed significantly more BIC compared to all other groups at 4 weeks. However, the only significant difference in BIC at 8 weeks was found between the lower CS group and the control group. The differences in BV density and ISQ value were not statistically significant at both 4 and 8 weeks. In addition, a higher concentration of CS and the application of sulfate hyaluronan exhibited no increase in BIC. It was concluded that the coating of extracellular matrix exhibited no beneficial effect in the aspect of BV density and ISQ value.

In the tibiae of rabbits, Lee et al. [57] studied whether implant surfaces coated with HA and type I collagen influence peri-implant bone formation. Four different implant surfaces were prepared and tested in 12 New Zealand rabbits: (1) uncoated control (acid-etched); (2) HA coated; (3) collagen + HA coated; and (4) collagen + HA + BMP-2. Peri-implant bone formation and BIC were assessed by histomorphometric analysis 6 weeks after implant placement. Under the histomorphometric analysis, only the collagen + HA coating surfaces displayed significantly greater peri-implant bone formation and BIC. Furthermore, adding BMP-2 to the implant surface did not show any advantage compared to the collagen + HA coating surface. It was concluded that collagen and HA coatings significantly increased new bone formation and BIC compared to the other coatings, and the additional BMP-2 coating did not display further benefits.

## Discussion

The primary intention of endosseous implant surface modifications is to absolutely modulate the implant and host tissue response and accomplish better osseointegration. This review evaluated whether bioactive surface modifications are capable of enhancing osseointegration in comparison to uncoated titanium surfaces in animals. This review has identified 34 research articles that used histomorphometric analysis to evaluate peri-implant bone formation in animals. Four categories of biomolecular coatings, including BMP, non-BMP growth factors, peptides, and ECM, were established in this review. Several different animal models were used in these 34 studies, suggesting different dynamics of bone formation especially in early healing times [58]. These factors may influence for the observed BIC values.

Four out of 8 BMP coating studies reported a positive effect on implant osseointegration [25, 26, 30, 31]. Among those 4 studies, two studies demonstrated a positive effect only with higher concentrations of BMP. Five out of 8 of the other growth factor coating studies reported an encouraging effect on peri-implant bone formation [32–34, 37, 39]. Whereas, 4 out of 5 studies in the peptide coating category exhibited a promising effect on osseointegration [40, 42–44]. Among those 4 studies, two of them reported a positive effect only at the early healing stage. Finally, there were 7 out of 13 studies for the ECM coating group that revealed a positive effect on bone formation. Two of the 7 studies showed that osseointegration was enhanced when two types of ECM were coated on implant surfaces. Therefore, bioactive surface modifications on implant surfaces do not always offer a beneficial effect on osseointegration. This review is partially in line with the systematic review and meta-analysis conducted by Jenny et al. [59]

One reason for the observed BIC variances in different animals could be the overall faster bone regeneration in dogs compared to pigs [59]. Therefore, the observed BIC may not reflect the actual effect of the specific type of surface modification on osseointegration in different animal models. Another factor that may influence peri-implant bone formation is the anatomic location of the implant placement as the dynamics of bone formation differ in different locations. It was reported that the percentage of BIC for implants coated with growth factors increased in extraoral locations and decreased in intraoral ones [59]. Furthermore, the time of healing in different animals may also play a critical role in the aspect of osseointegration. Implant healing time showed large variation ranging from 1 week to 6 months. However, some studies assessed a single time point, while others evaluated up to four time points.

In summary, the present review assessed the impact of bioactive surface modifications on implant osseointegration. It was difficult to conclude that biological implant surface coating offers a beneficial effect on peri-implant bone formation. Readers must keep in mind that all these factors make the inter-study comparison more difficult and further complicate the interpretation of results. Furthermore, long-term prospective clinical studies in humans are required to verify whether a positive effect in animals will translate to humans.

## Conclusions

Bioactive surface modifications on implant surfaces do not always offer a beneficial effect on osseointegration. Nevertheless, surface modifications of titanium dental implants with biomolecular coatings seem to promote peri-implant bone formation, resulting in enhanced osseointegration during the early stages of healing. Several factors make the inter-study comparison more difficult and further complicate the interpretation of results in peri-implant bone formation. However, long-term clinical studies are needed to validate the long-term success of biomolecular coated implants. Furthermore, it must be kept in mind that results of animal experiments need not necessarily reflect the human clinical reality.

## Abbreviations

APC: Anodic plasma-chemical
BIC: Bone to implant contact
Bisphos: Bisphosphonate-coated
BMPs: Bone morphogenetic proteins
BVD: Bone volume density
CaP: Calcium phosphate
CaP/BMP-2 ads + inc: Bone morphogenetic protein-2 adsorbed to and incorporated into calcium phosphate-coated titanium surfaces
CaPTi: Calcium phosphate-coated titanium surfaces
CaPTi/BMP-2 ads: Bone morphogenetic protein-2 adsorbed to calcium phosphate-coated titanium surfaces
CaPTi/BMP-2 inc: Bone morphogenetic protein-2 incorporated into calcium phosphate-coated titanium surfaces
Coll: Collagen type I
Coll/CS: Collagen type I/chondroitin sulfate
Coll/CS/BMP: Collagen/chondroitin sulfate/bone morphogenetic protein
Coll/CS/BMP-4: Collagen type I/chondroitin sulfate/bone morphogenetic protein-4
Coll/CS/DC/BMP/TGF: Collagen/chondroitin sulfate/decorin/ bone morphogenetic protein/ transforming growth factor
Coll/CS/rhBMP-4: Collagen/chondroitin sulfate/bone morphogenetic protein-4
Coll/DC: Collagen/decorin
Coll/DC/TGF: Collagen/decorin/transforming growth factor
ColpTi: Anodized titanium surfaces with collagen
CSA: Chromosulfuric acid
DAE: Dual acid-etched
ECM: Extracellular matrix
FGF-FN: Fibroblast growth factor-fibronectin
HA: Hydroxyapatite
MS: Machined surface
Pi: Push-in type
PLGA: Poly(lactide-co-glycolide)
PLL-g-PEG: Poly(L-lysine)-graft-poly(ethylene glycol)
PRGF: Plasma-rich growth factor
rBVD: Relative peri-implant bone volume density
RDG: (Arg-Asp-Gly) peptide
RFA: Resonance frequency analysis
RGD: (Arg-Gly-Asp) peptide
rhbFGF: Recombinant human basic fibroblast growth factor
rhBMP-2: Recombinant human bone morphogenetic protein-2
rhIGF-1: Recombinant human insulin-like growth factor-1
SLA: Sandblasted and acid-etched
St: Screw type
TGF-β1: Transforming growth factor β1
Ti: Titanium surfaces
Ti/BMP-2 ads: Bone morphogenetic protein-2 adsorbed to uncoated titanium surfaces
TPO: Titanium porous oxide
Zr: Zirconia
